# Non-Cell-Autonomous Regulation of Retrograde Motoneuronal Axonal Transport in an SBMA Mouse Model

**DOI:** 10.1523/ENEURO.0062-16.2016

**Published:** 2016-08-10

**Authors:** Katherine Halievski, Michael Q. Kemp, S. Marc Breedlove, Kyle E. Miller, Cynthia L. Jordan

**Affiliations:** 1 Neuroscience Program, Michigan State University, East Lansing, Michigan 48824-1115; 2 Department of Integrative Biology, Michigan State University, East Lansing, Michigan 48824-1115; 3 Department of Physiology, Michigan State University, East Lansing, Michigan 48824-1115

**Keywords:** androgen, androgen receptor, Kennedy’s disease, neuromuscular disease, skeletal muscle, testicular feminization mutation

## Abstract

Defects in axonal transport are seen in motoneuronal diseases, but how that impairment comes about is not well understood. In spinal bulbar muscular atrophy (SBMA), a disorder linked to a CAG/polyglutamine repeat expansion in the androgen receptor (*AR*) gene, the disease-causing AR disrupts axonal transport by acting in both a cell-autonomous fashion in the motoneurons themselves, and in a non-cell-autonomous fashion in muscle. The non-cell-autonomous mechanism is suggested by data from a unique “myogenic” transgenic (TG) mouse model in which an AR transgene expressed exclusively in skeletal muscle fibers triggers an androgen-dependent SBMA phenotype, including defects in retrograde transport. However, motoneurons in this TG model retain the endogenous *AR* gene, leaving open the possibility that impairments in transport in this model also depend on ARs in the motoneurons themselves. To test whether non-cell-autonomous mechanisms alone can perturb retrograde transport, we generated male TG mice in which the endogenous *AR* allele has the testicular feminization mutation (*Tfm*) and, consequently, is nonfunctional. Males carrying the *Tfm* allele alone show no deficits in motor function or axonal transport, with or without testosterone treatment. However, when Tfm males carrying the myogenic transgene (Tfm/TG) are treated with testosterone, they develop impaired motor function and defects in retrograde transport, having fewer retrogradely labeled motoneurons and deficits in endosomal flux based on time-lapse video microscopy of living axons. These findings demonstrate that non-cell-autonomous disease mechanisms originating in muscle are sufficient to induce defects in retrograde transport in motoneurons.

## Significance Statement

Our findings suggest that therapies targeting skeletal muscle could potentially rescue motoneurons from axonal transport dysfunction in neuromuscular disease. Axonal transport is critical for proper motoneuronal functioning and is often impaired in neurodegenerative disease, including spinal bulbar muscular atrophy, an androgen-dependent neuromuscular disease linked to a polyglutamine expansion in the androgen receptor. In this study, we show that AR activated by androgens exclusively in skeletal muscle is sufficient to trigger defects in retrograde transport in the motoneurons. Specifically, diseased mice show impaired retrograde labeling of motoneurons *in vivo* and defective endosomal transport in living axons *ex vivo.* Thus, one trait of diseased motoneurons, impaired axonal transport, can be conferred by disease processes originating in muscle.

## Introduction

Projection neurons are particularly susceptible to defects in axonal transport due to their long processes (De Vos et al., 2008; Morfini et al., 2009). This has led to an interest in the role of axonal transport in neurodegenerative diseases in which projection neurons are affected, including motoneurons in spinal bulbar muscular atrophy (SBMA), a progressive neuromuscular disease linked to a CAG expansion mutation in the androgen receptor (*AR*) gene (La Spada et al., 1991). SBMA occurs only in men and is androgen dependent (Katsuno et al., 2012; Baniahmad, 2016). With only one exception (Malik et al., 2011), studies of axonal transport in both SBMA models and SBMA patients suggest that this key neuronal process is perturbed and may be an early event in disease (Piccioni et al., 2002; Szebenyi et al., 2003; Katsuno et al., 2006; Morfini et al., 2006; Kemp et al., 2011). How AR disrupts axonal transport, however, is not clear. Evidence suggests that the disease-causing AR can act directly in the motoneuron in a cell-autonomous fashion to disrupt axonal transport (Szebenyi et al., 2003) and/or indirectly in a non-cell-autonomous manner via the target musculature (Kemp et al., 2011). Understanding where and how AR impairs axonal transport and whether perturbed transport contributes to motor dysfunction in SBMA is critical for developing effective treatments for this disease.

That non-cell-autonomous mechanisms can instigate motoneuronal dysfunction in neuromuscular disease has been the subject of several recent reviews (Jordan and Lieberman, 2008; Ilieva et al., 2009; Sambataro and Pennuto, 2012). Because motoneurons depend on their targets to survive in development (Hamburger and Levi-Montalcini, 1949) and to function normally in adulthood (Navarro et al., 2007), it stands to reason that disease-related dysfunction originating in muscle could trigger dysfunction in the motoneurons. However, whether axonal transport is susceptible to such non-cell-autonomous influences has not been firmly established. In these terms, because both motoneurons and muscle function are impaired in disease models of SBMA, they provide an excellent model system for testing whether defects in axonal transport can arise non-cell autonomously.

Reports indicate that retrograde transport is impaired in an androgen-dependent fashion, paralleling the loss of motor function, in a myogenic model of SBMA (Kemp et al., 2011). This unique myogenic mouse model expresses transgenic (TG) wild-type (WT) ARs at high levels only in skeletal muscle. While these data imply that signals originating in muscle can impair retrograde axonal transport in motoneurons, because AR is also expressed throughout the body in TG animals by virtue of the endogenous *AR* gene, it leaves open the possibility that the effect of androgen on motoneuronal retrograde transport may have also depended on ARs acting in other cell types, including the motoneurons themselves. To eliminate this possibility, we generated mice in which the only source of functional ARs was from the transgene that is expressed only in muscle. We did this by passing the transgene into males carrying an endogenous allele for AR that is dysfunctional, the testicular feminization mutation *(Tfm*), providing a null background for functional ARs. Genetic males carrying the *Tfm* allele are insensitive to androgens, exhibiting a female phenotype, but have normal motor function (Zuloaga et al., 2008; Johansen et al., 2011). Thus, male mice carrying both the *Tfm* allele of the endogenous *AR* gene, and the AR transgene with its expression controlled by the muscle-specific human skeletal α-actin promoter (Tfm/TG), have functional ARs only in a single cell type—skeletal muscle fibers. This strategy allowed us to ask whether a disease-causing *AR* allele expressed solely in muscle fibers is sufficient to impair axonal transport in motoneurons via a non-cell-autonomous mechanism.

Using cholera toxin (CT) to label retrogradely transporting endosomes, we found fewer labeled motoneurons in the lumbar spinal cord of motor-impaired Tfm/TG males than in the lumbar spinal cord of Tfm-only control males with intact motor function, a deficit in Tfm/TG males that is comparable to prior results in myogenic TG males on a background of WT AR (Kemp et al., 2011). Analysis of retrogradely trafficking endosomes in living axons of diseased Tfm/TG male mice also revealed the same deficit in endosomal flux as found previously (Kemp et al., 2011). The present data confirm that non-cell-autonomous mechanisms originating in target muscle fibers of motoneurons can perturb retrograde axonal transport in the motoneurons innervating them.

## Materials and Methods

### Tfm/TG mouse model

Tfm/TG mice that express functional AR only in skeletal muscle fibers were generated by *in vitro* fertilization. Sperm was harvested for *in vitro* fertilization from a TG male that overexpresses WT ARs specifically in muscle cells (Monks et al., 2007). Female *Tfm* carriers on a C57BL/6 J background were superovulated, and eggs were harvested. The *Tfm* allele renders AR protein nonfunctional and causes androgen-insensitivity uniformly throughout the body (He et al., 1991). After fertilization, embryos were transferred into pseudopregnant recipient B6D2F1/J mice. Four founding females were produced that carried both the transgene and the *Tfm* allele. Female offspring carrying both the *Tfm* gene and the AR transgene were mated to WT C57BL/6 males to produce the experimental Tfm/TG males.

Many Tfm/TG males die at birth, as do TG males with a WT AR background (Monks et al., 2007). This perinatal lethality is caused by prenatal exposure to endogenous testicular androgens. To enhance perinatal survival of such Tfm/TG males, pregnant dams were treated with the AR antagonist flutamide. Pregnant Tfm/TG carrier females were injected subcutaneously with 5 mg flutamide in propylene glycol at the nape of the neck from gestational day 15 to 20 (day of mating was designated as day 0). Control males used in this study were littermate males that carried only the *Tfm* allele (Tfm males) and, thus, were exposed to the same prenatal flutamide treatment as Tfm/TG males. Because Tfm male mice develop a deficit in androgen production postnatally (Goldstein and Wilson, 1972; Murphy and O'Shaughnessy, 1991), Tfm/TG males have low, female-like levels of circulating androgens as adults, comparable to Tfm males (Johansen et al., 2011). This means that postnatally Tfm/TG males are asymptomatic (like TG females) and express disease symptoms only when treated with exogenous testosterone (T) to increase T levels to that of normal males (Johansen et al., 2009). All animal procedures were performed in accordance with the regulations of the Michigan State University Animal Care Committee.

### Adult hormone treatment to induce disease symptoms

To induce disease symptoms in adult Tfm/TG males, 150-d-old Tfm/TG and Tfm male littermates under isoflurane anesthesia received subcutaneous implants at the nape of the neck just below the interscapular fat pad of Silastic capsules containing crystalline T (inner diameter, 1.57 mm; outer diameter, 3.18 mm; 6 mm, effective release length). Such T implants result in serum T levels comparable to that of adult gonadally intact males (Johansen et al., 2009). After 5 d of treatment, the efficiency of retrograde transport was assessed in the following two different ways (as detailed below): retrograde labeling of motoneuronal cell bodies with horseradish peroxidase (HRP) injected into muscle, and active transport of fluorescently labeled endosomes in living axons of the sciatic nerve. In each case, the label was conjugated to cholera toxin (CT). Forelimb grip strength was monitored using a grip strength meter (Columbus Instruments) in Tfm/TG males and their Tfm male brothers during androgen treatment, beginning just prior to implanting T capsules (day 0), and then on days 1, 2, 3, 4, and 5 of treatment. Data are reported in the figure captions as means and standard error of the mean (SEM), and *N* is the number of animals per group.

### Retrograde labeling of spinal motoneurons

Spinal motoneurons were retrogradely labeled by injecting CT B conjugated to HRP (CT-HRP; 1 µl of 0.2% CT-HRP/muscle; List Biological Laboratories) into the anterior tibialis (AT) muscles of isoflurane anesthetized mice. Once mice recovered from anesthesia, they were returned to their home cage. After 12 h to allow CT-HRP to retrogradely transport to motoneuronal cell bodies, mice were reanesthetized with an intraperitoneal injection of sodium pentobarbital. Once deeply anesthetized, mice were perfused with 0.9% saline followed by cold 0.1 m phosphate buffer (pH 7.4) containing 0.8% paraformaldehyde and 1.25% glutaraldehyde. Spinal cords were dissected out and placed in the same fixative for 5 h at 4°C, after which they were transferred to a 10% phosphate-buffered sucrose solution and held overnight at 4°C. The following day, spinal cords were transversely sectioned on a freezing, sliding microtome at 40 μm. Sections were reacted with tetramethylbenzidine (TMB) for histological visualization of HRP (Park et al., 2002). Sections were washed with distilled water and incubated with a mixture of 0.005% TMB and 0.1% sodium cyanide for 20 min. H_2_O_2_ was added for 20 min, and the reaction was stopped by washing in 0.01 m sodium acetate buffer. Alternate sections were mounted on gelatin-coated slides within 6 h after TMB reaction. The next day, the sections were dehydrated, defatted, and coverslipped. Slides containing TMB-reacted sections were stored in opaque boxes at 4°C to prevent degradation of TMB reaction product. All slides were coded and analyzed without knowledge of treatment groups. Estimates of the number of retrogradely labeled AT motoneurons were based on total bilateral counts performed on adjacent sections at 100× magnification. Every lumbar section was examined for labeled motoneurons, including several sections beyond the rostral and caudal extent of AT motoneurons. Data are reported in the figure captions as overall means and SEM, and *N* is the number of animals per group.

### Live imaging of endosomal transport in sciatic nerve axons

Endosomal trafficking in sciatic nerve axons of diseased (T-treated Tfm/TG) and healthy (T-treated Tfm) male mice was assessed by directly monitoring the retrograde transport of endosomes in living axons. CT B conjugated to Alexa Fluor 488 (CT-AF488, Molecular Probes; 10 µl of 0.2% in 0.9% saline containing 1% DMSO) was injected into the AT and gastrocnemius muscles of isoflurane-anesthetized mice to label endosomes. Retrograde movement of endosomes in living axons of explanted sciatic nerves was monitored 4 h after injection. During the 4 h delay, mice were awake and mobile.

Mice were re-anesthetized with isoflurane to harvest the sciatic nerve. Explants (∼10 mm in length) were placed on coverglass, glued in place (Vetbond, 3M), and covered with 37°C oxygenated bicarbonate Ringer’s solution (in mm: 135 NaCl, 5 KCl, 1 MgCl_2_, 1.5 NaHCO_3_, 1 Na_2_PO_4_, 2 CaCl_2_, and 1.1 glucose, pH 7.2). The right sciatic nerve was harvested first and recordings were made from it, while the left nerve remained *in situ* in the anesthetized mouse. There is no evidence that transport in left and right nerves systematically differs or that transport declines over time (data not shown), which is consistent with previous findings (Kemp et al., 2011). Body temperature was maintained using a heating pad for the 30 min between nerve harvests. Once both nerves were harvested, the mouse was killed.

### Live image acquisition and processing

Time-lapse movies of moving endosomes were made using an inverted LiveScan swept field confocal microscope (Eclipse TE2000-E, Nikon) equipped with a 60× PlanApo oil-immersion objective (1.4 numerical aperture), illuminated with the 488 nm line of a 150 mW argon laser (Melles Griot), and recorded with a Photometrics CoolSNAP HQ2 camera using NIS Elements software. Temperature of the sciatic nerve explant was maintained at 37°C by a ring incubator. Less than 10 min after nerve harvest, recordings began, with two time-lapse movies taken from the same explant within 30 min of harvest. Each movie was 7 min in length, comprising 211 images, with images captured every 2 s at a 1 s exposure. Laser power and aperture settings were identical for all captured images. Scanning for trafficking started at the center of the sciatic nerve segment and proceeded toward the distal end of the nerve to reduce variability. Because of concerns about photobleaching, different axons in the same explants were used for the two movies, and the second movie was taken from a region distal to the first. To minimize experimenter bias, video capture began when the first moving endosome could be kept in focus.

### Trafficking measures from kymographs

**Figure 1.**
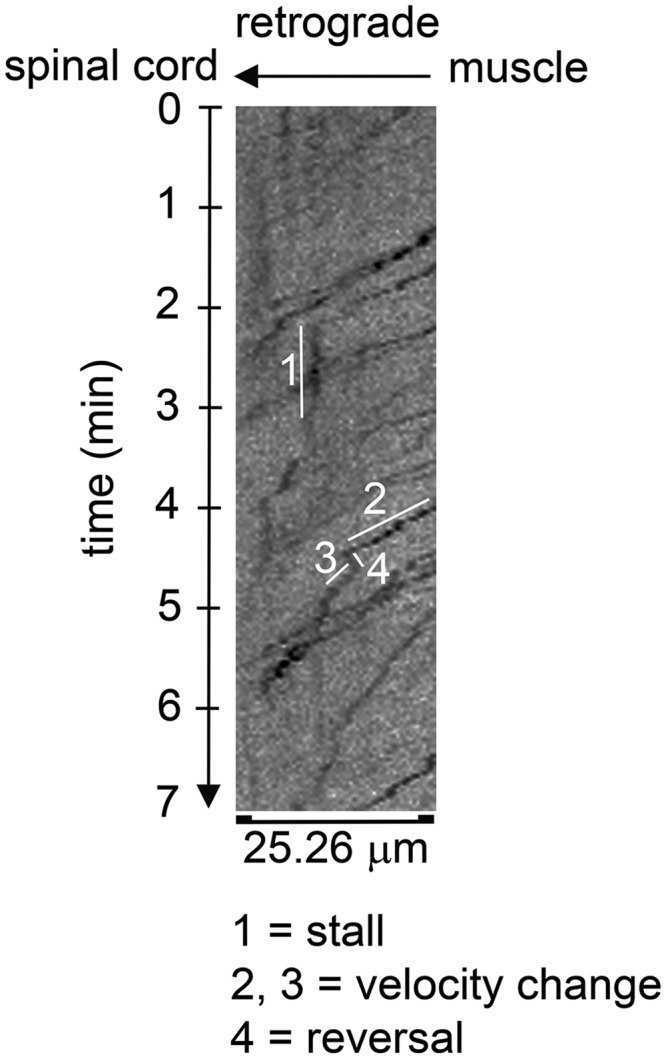
Representative kymograph showing various trafficking perturbations measured. Endosomal traces are seen as dark irregular lines that typically slope downward from right to left, indicating the time it takes for a given endosome to traverse a given length (25.26 μm) of axon. Note that the slope of the trace can change over time, going from a downward slope to one that is vertical (parallel to the time axis) indicating a stall (1), to less severe changes in slope (2 vs 3), with the steeper downward slope (3) indicating a transient increase in endosomal velocity. A negative slope in an endosomal trace (4) indicates that the endosome transiently reversed its direction of movement. The shown kymograph is taken from a healthy Tfm male.

Time-lapse movies were converted into kymographs, single two-dimensional images showing the movement of endosomes (referred to as “endosomal traces”) along the length of an axon as a function of time (Fig. 1). To make kymographs, the resulting movies were opened in NIH ImageJ as a 16 bit stack of images then rotated 90° counterclockwise. A 60-pixel-wide length of axon was then cropped, and using the “re-slice” option, sliced into 1-pixel-width sections and *z*-projected using the SUM option, which resulted in 32 bit “raw” kymographs. The raw kymographs were converted to 16 bits, and saved as tiff files. To facilitate the visibility of transport events, raw kymographs were opened in Adobe Photoshop (version 7.0 or CS2), color inverted, and resampled to increase size by 500%, thus producing the kymographs used for analysis, using ImageJ. We measured flux, net, and instantaneous velocity, run length, and frequency of trafficking perturbations (stalls, reversals, and velocity changes) for retrogradely transporting endosomes.

Endosomal flux is the number of retrogradely transporting endosomes in an axon per unit of time and was determined by drawing a line through the center of the kymograph parallel to the time axis and counting the number of endosomal traces that cross that line. The total number of endosomes per kymograph was then divided by 7 min (total time represented on the kymograph) to estimate endosomal flux as the number of endosomes per minute. Such estimates were averaged across individual axons within a single animal to obtain a single estimate of flux per mouse. Net velocity of retrogradely transported endosomes is the net distance traveled by each endosome in the distal to proximal direction over time and included stalls, reversals in direction, and/or changes in velocity that might have occurred. Measures of net velocity were obtained from kymographs by drawing a box around each endosomal trace such that the far ends of the trace pass through opposite corners of the box. The height and width of the box (in pixels) corresponding to length and time are used to calculate velocity (in microns per second) based on the following formula: [distance (pixels) × 0.10526 (microns per pixel)/time (pixels) × seconds per frame]. Net velocities of individual endosomal traces in a single kymograph were then averaged to provide the mean estimate of the net velocity per axon. These estimates were then averaged across individual axons sampled within a single animal to obtain a single estimate of net velocity per mouse.

Instantaneous velocity was measured by drawing a 1-pixel-wide line parallel to the time axis, bisecting the kymograph with respect to distance. For each endosomal trace crossing the center line, a 20 pixel box was drawn around it, centering it with respect to the bisecting line, with width and height of the box representing distance and time, respectively. The width of the box (20 pixels) accounted for 6.6% (1.67 mm) of the total distance traveled, and any continuous velocity within it was considered instantaneous. The time it took to travel this short distance was calculated based on the height of the box, which was adjusted so that its corners intersected the most distal and proximal aspects of the trace. On rare occasions, perturbations in endosomal velocity, including changes in speed, stalls, and/or reversals, appeared within the box. In such cases, the box was moved immediately distal to the trafficking perturbation or until the box included a part of the endosomal trace that contained no trafficking perturbations. Instantaneous velocity (in microns per second) was calculated based on the same formula used for net velocity. Measures of instantaneous velocity for each endosome were averaged within a single kymograph, and such estimates from two to four axons of the same mouse were averaged to obtain a single estimate of instantaneous velocity per mouse.

We also assessed the frequency of trafficking perturbation events, which reflects the efficiency by which endosomes are transported retrogradely. We counted the number of trafficking perturbation events per trace, which included changes in velocity, stalls, and/or reversals that lasted for ≥2 s (Fig. 1). Our measure of velocity changes was based on visible changes in velocity in the kymograph without directly measuring changes in the slope of the endosomal trace, indicating that subtle changes in velocity may have not been detected. Stalls were defined as an endosome that was stationary for ≥2 s. Stalling prevalence was assessed by determining the number of stalls per endosomal trace and the proportion of traces that had stalls. We also measured average run length, which was estimated by adding together the total distance of endosomal transport observed in a kymograph and dividing this by the number of stalls. Data for reversals, stalls, velocity changes, and overall trafficking perturbations are presented as a percentage of total endosomes. Mean estimates of trafficking measures were based on 15–30 endosomal traces per kymograph, with two to four kymographs per mouse, where *N* is the number of animals/group. Analyses of kymographs were performed by an experimenter blind to animal identification and genotype. Refer to the statistical table (Table 1) for a description of the data structure, the statistical test used, and 95% confidence intervals of data.

**Table 1:** Statistical table

	Data structure	Type of test	95% Confidence interval for the mean
^a^Grip strength	Normal	Repeated-measures ANOVA	Day 0: n/a
			Day 1: Tfm (84.72, 136.78); Tfm/TG (37.74, 79.06)
			Day 2: Tfm (69.78, 124.22); Tfm/TG (15.43, 49.37)
			Day 3: Tfm (60.88, 142.62); Tfm/TG (10.06, 30.34)
			Day 4: Tfm (82.57, 118.43); Tfm/TG (−6.59, 25.39)
			Day 5: Tfm (74.99, 113.01); Tfm/TG (−8.81, 31.61)
^b^HRP filled motoneurons	Normal	Independent *t* test	Tfm (93.81, 195.19); Tfm/TG (51.44, 90.96)
^c^Flux	Normal	Independent *t* test	Tfm (3.49, 8.16); Tfm/TG (2.22, 4.52)
^d^Net velocity	Normal	Independent *t* test	Tfm (0.25, 0.53); Tfm/TG (0.30, 0.45)
^e^Instantaneous velocity	Normal	Independent *t* test	Tfm (0.41, 0.86); Tfm/TG (0.34, 0.58)
^f^Overall trafficking perturbations	Normal	Independent *t* test	Tfm (3.46, 18.79); Tfm/TG (10.52, 34.11)
^g^Velocity changes	Normal	Independent *t* test	Tfm (1.12, 5.61); Tfm/TG (2.86, 11.61)
^h^Reversals	Normal	Independent *t* test	Tfm (3.10, 18.79); Tfm/TG (11.20, 34.10)
^i^Stalls	Normal	Independent *t* test	Tfm (0.75, 8.92); Tfm/TG (4.53, 16.11)
^j^Run length	Tfm group positively skewed (skewness, 2.198)	Independent *t* test	Tfm (−444.43, 1881.98); Tfm/TG (41.77, 499.35)

Structure of data, statistical test used, and 95% confidence intervals are listed for each variable measured.

## Results

**Figure 2.**
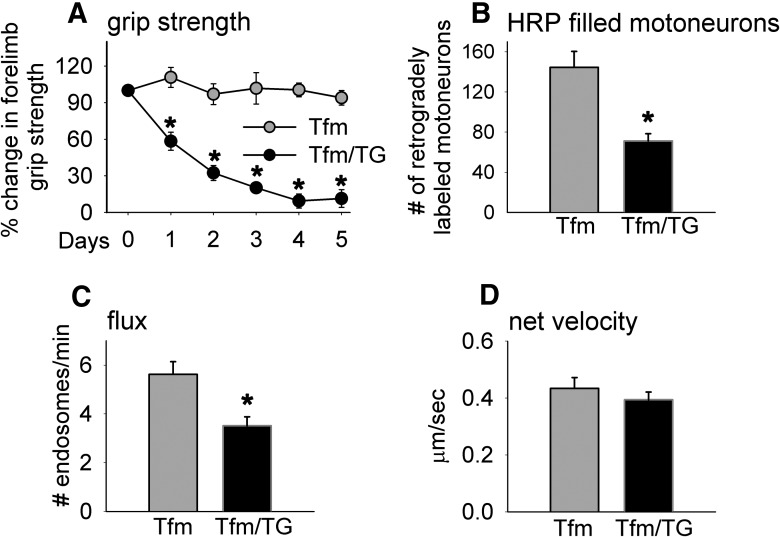
Adult male mice with functional ARs only in skeletal muscle fibers (Tfm/TG) show defects in motor function, retrograde transport, and endosomal trafficking. ***A***, Myogenic TG male mice with a mutant allele (*Tfm*) of the endogenous *AR* gene (Tfm/TG) show an androgen-dependent loss of grip strength, with grip strength dropping to basement within 4 d of T exposure. Both groups of males were treated with exogenous T, given their negligible levels of endogenous androgen. Note that the grip strength of Tfm controls that lack functional ARs is unaffected by T administration. ***B***, The number of anterior tibialis motoneurons filled with CT-HRP after 12 h of retrograde transport is significantly reduced in diseased Tfm/TG males compared with healthy Tfm controls, indicating that transgenic AR acting in muscle fibers induces defects in retrograde transport, independent of endogenous wild-type AR in motoneurons and elsewhere. ***C***, ***D***, Such Tfm/TG mice show deficits in flux, but not net velocity, after 5 d of T treatment, which is comparable to reported results from TG males on a wild-type background (Kemp et al., 2011). The current data reinforce the idea that muscle AR instigates a disease process that retrogradely impairs endosomal trafficking in motoneurons, possibly by perturbing aspects of the early endocytotic pathway. Graphs represent the mean ± SEM (***A***, ***B***, *N* = 4-5/group; ***C***, ***D***, *N* = 6/group). A repeated-measures ANOVA indicated a grip strength × group interaction, and further revealed an effect of time on grip strength only for the Tfm/TG genotype. *Significant differences (*p* < 0.05) between groups based on a one-way ANOVA (***A***) and independent *t* test (***B–D***).

We find that motor function based on the forelimb grip strength of adult Tfm/TG males rapidly declines during the 5 d of androgen treatment, while motor function in Tfm-only males remains stable and is unaffected by androgen (Fig. 2*A*). After only 24 h of androgen exposure, motor function in Tfm/TG males is significantly reduced compared with Tfm-only males (day 1, *p* = 0.002) and continues to drop progressively during the next 4 d (day 2, *p* = 0.0004; day 3, *p* = 0.0003; day 4, *p* = 0.00001; day 5, *p* = 0.00006)^a^, exhibiting the same time course of demise as seen in T-treated myogenic TG females (Johansen et al., 2009). These data also replicate previous results for Tfm/TG males (Johansen et al., 2011). Both Tfm and Tfm/TG males without T treatment have T levels that are ∼10 times lower than those of adult WT males (Johansen et al., 2011), suggesting that the low circulating level of T is why adult Tfm/TG males do not develop motor dysfunction on their own.

Muscles of Tfm/TG mice and Tfm controls were injected with CT-HRP at the end of treatment (5 d) to assess the efficiency of transport based on the number of retrogradely labeled motoneurons. We find that motor-impaired Tfm/TG males have a significant deficit in the number of retrogradely labeled motoneurons compared with Tfm controls (Fig. 2*B*; *p* = 0.0027)^b^. Interestingly, the number of labeled motoneurons for Tfm control males is comparable with that previously reported for gonadectomized WT males, who lack testicular androgens (Kemp et al., 2011), with the number of retrogradely labeled motoneurons somewhat reduced compared with those of gonadally intact WT males. It is possible that one of the normal roles of WT AR when activated by endogenous androgens is to enhance the retrograde transport of cargo to motoneuronal cell bodies.

We also find that endosomal transport in the axons of diseased Tfm/TG males was affected when assessed directly in living axons *ex vivo* using video microscopy. Specifically, endosomal flux (*p* = 0.0079)^c^, but not net velocity (*p* = 0.411)^d^, is significantly impaired in sciatic nerve axons (Fig. 2*C*,*D*), suggesting that deficits in flux may account for the reduced number of retrogradely labeled motoneurons in diseased Tfm/TG mice. This pattern of results is also seen in both chronically diseased myogenic TG males and acutely diseased myogenic TG females (Kemp et al., 2011).

**Figure 3.**
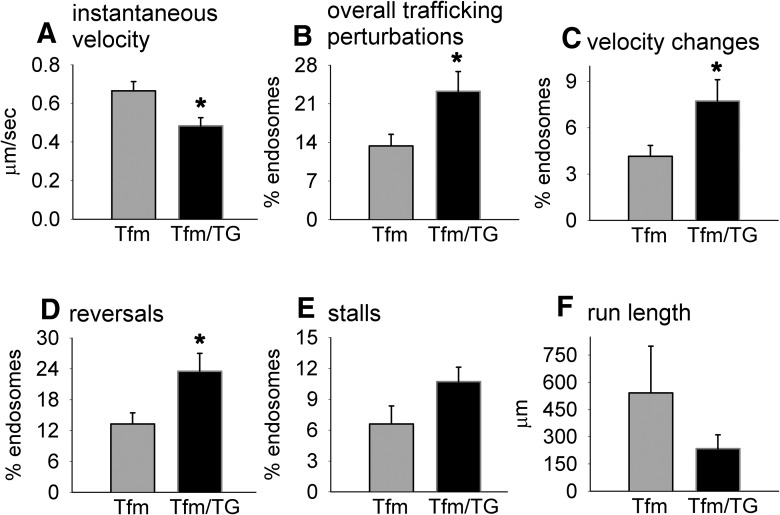
Androgen-activated AR in skeletal muscle fibers perturbs trafficking kinetics of retrogradely transporting endosomes. ***A***, Based on live imaging of fluorescently labeled trafficking endosomes in sciatic nerve axons of Tfm and Tfm/TG males, we find that the instantaneous velocity of transporting endosomes is significantly decreased in diseased Tfm/TG males compared with Tfm control males. ***B–E***, Endosomes in motor-impaired Tfm/TG males also show elevated levels of trafficking perturbations overall (***B***), including an increased tendency to show velocity changes (***C***), reversals (***D***), and stalls (***E***). ***F***, The average run length of retrogradely transporting endosomes was also shorter in diseased Tfm/TG males, but this difference was not significant, perhaps due to the larger error variance in run length for Tfm males. Because Tfm and Tfm/TG normally have very low levels of circulating androgens, males used in this study were treated with testosterone, mimicking normal male levels of androgens. Such trafficking perturbations demonstrate that non-cell-autonomous mechanisms from skeletal muscle can impair trafficking machinery in the axon proper, possibly perturbing the retrograde motor dynein and its associated dynactin complex. Graphs represents the mean ± SEM (***A–F***, *N* = 6/group) and significant differences (*p < 0.05) between groups based on an independent *t* test.

We also measured instantaneous velocity and trafficking perturbations and unexpectedly detected a deficit in instantaneous velocity (Fig. 3*A*; *p* = 0.0178)^e^, an outcome that was not apparent in myogenic TG males (Kemp et al., 2011). These data suggest that endogenous WT-AR may have protected against the effects of a toxic muscle AR on instantaneous velocity. Altered instantaneous velocity in Tfm/TG males suggests defects in dynein function, a notion that is not without precedence, inasmuch as another SBMA mouse model showed reduced dynein heavy chain levels in the ventral spinal root (Katsuno et al., 2006). We also find that a greater percentage of endosomes show trafficking perturbations in diseased Tfm/Tg males (Fig. 3*B*; *p* = 0.0387)^f^, which is caused by more endosomes in diseased Tfm/TG males showing changes in velocity (Fig. 3*C*; *p* = 0.0435)^g^ and reversals (Fig. 3*D*; *p* = 0.0314)^h^ compared with endosomes in the axons of healthy Tfm males. Surprisingly, although more endosomes tend to stall in diseased axons than in healthy axons, this difference was not significant (Fig. 3*E*; *p* = 0.0955)^i^. Likewise, while the mean run length of endosomes in Tfm/TG is shorter, it too was not significantly decreased compared with that of healthy Tfm males (Fig. 3*F*; *p* = 0.2784)^j^. Together, these data suggest that endogenous WT ARs may have protected against some of the effects of a toxic muscle AR on transport, and converge on the conclusion that AR acting only in muscle fibers is sufficient to impair retrograde transport/trafficking of endosomes in motoneurons.

## Discussion

Here we demonstrate that retrograde axonal transport in motoneurons is impaired via non-cell-autonomous AR action in muscle. Myogenic contributions to SBMA were first recognized in two different mouse models (Yu et al., 2006; Monks et al., 2007), where myopathic changes were discovered in a knock-in model well before neuropathy and transgenically overexpressed WT-AR specifically in muscle cells triggered SBMA-like symptoms only in myogenic TG males, including a loss of ventral root axons. These unexpected findings suggested that non-cell-autonomous mechanisms originating in muscle could drive pathogenesis in motoneurons in SBMA mice. We now find that AR in muscle is sufficient to disrupt retrograde transport in the motoneurons that innervate diseased muscle. Previous findings indicated that myogenic TG mice exhibit perturbed axonal transport compared with WT littermates (Kemp et al., 2011). However, such TG mice also had AR in other cell types due to expression of the endogenous gene, leaving open the possibility that the transport deficits found in this model also depended on endogenous *AR*. Thus, we directly addressed this question by assessing axonal transport in myogenic TG males that also have the *Tfm* allele of the endogenous *AR* gene (Tfm/TG males), thus eliminating all functional AR from the endogenous gene. Consequently, such Tfm/TG males had functional AR only in skeletal muscle fibers due to the expression of the AR transgene. We found that diseased Tfm/TG males showed comparable deficits in the number of retrogradely labeled motoneurons and in endosomal flux as previously reported for diseased TG males on a WT AR background (Kemp et al., 2011), indicating that AR acts solely in muscle fibers to perturb retrograde transport in motoneurons.

As expected, Tfm/TG males exhibited androgen-dependent motor dysfunction, replicating a previous finding from this same mouse model showing that such dysfunction is fully reversible when androgen treatment ceases (Monks et al., 2007; Johansen et al., 2009, 2011; Fig. 2*A*) and is consistent with the phenotype of SBMA mouse models expressing a CAG-expanded *AR* allele (Katsuno et al., 2002; Chevalier-Larsen et al., 2004; Sopher et al., 2004; Yu et al., 2006; Renier et al., 2014). Such mice also showed a striking deficit in the number of retrogradely labeled motoneurons compared to nondiseased Tfm male controls (Fig. 2*B*), correlating with the deficit in motor function (Fig. 2*A*). Interestingly, the magnitude (∼50%) of the labeling deficit in Tfm/TG males is comparable to that previously reported for both chronically diseased myogenic TG males and acutely diseased myogenic TG females (Kemp et al., 2011), indicating that TG AR in muscle, when activated by androgens, rapidly perturbs the uptake/retrograde transport of CT-HRP by motoneuronal axons independent of AR expressed by the native gene, including that in motoneurons and/or other cell types.

The deficit in axonal transport exhibited by androgen-treated Tfm/TG males is largely comparable to that previously observed in TG-only males and females. Notably, deficits in CT-HRP staining and grip strength are apparent after only 24 h of T treatment in TG-only females (Kemp et al., 2011), the time point at which Tfm/TG males, given the same T treatment, show deficits in grip strength. Moreover, CT-HRP transport recovers fully in chronically symptomatic TG males that have been castrated, as does motor function in symptomatic Tfm/TG males following T removal (Johansen et al., 2011; Kemp et al., 2011). It is clear that the transgene alone without androgen stimulation is not sufficient to induce transport dysfunction, in parallel with the effect of the transgene on motor function, since castrated TG males and untreated TG females show deficits in neither motor function nor axonal transport (Kemp et al., 2011). Unfortunately, any direct comparison between TG and Tfm/TG males in disease traits would be confounded by long-term versus short-term effects of disease. TG-only males are exposed throughout life to endogenous male levels of androgens, causing secondary effects of disease due to its chronic nature in TG-only males. On the other hand, Tfm/TG males, like TG-only females, lack endogenous male levels of androgens postnatally, and only express an acute disease phenotype when provided with exogenous androgens. In sum, the defects in transport and motor dysfunction induced by T treatment of Tfm/TG males, who, like TG-only males and females, express the disease-causing *AR* allele only in muscle fibers, is comparable to what is seen in TG-only males and females in which both motor function and axonal transport are disrupted only in the presence of male levels of androgens.

Evidence strongly argues that the deficit found in the number of retrogradely labeled motoneurons is not caused by a net loss of motoneurons. Cell counts of motoneuronal cell bodies in Nissl-stained sections of the spinal cord show no evidence of motoneuronal loss in the myogenic TG model (Monks et al., 2007; Johansen et al., 2009). Moreover, extending CT transport time to 24 h rather than the 12 h used here eliminates the deficit in retrograde labeling of motoneurons (Kemp et al., 2011). Notably, the defects in axonal transport observed here are not likely due to secondary effects of disease since deficits in CT-HRP transport occur just 24 h after the commencement of T treatment in female TG mice (Kemp et al., 2011) who display the same time course of deterioration as the inducible Tfm/TG model used here. Moreover, no muscle atrophy is apparent in TG females treated for 5 d, despite their displaying severe motor impairment and a loss of muscle force that does not involve a loss of muscle mass (Oki et al., 2013). Additionally, no dying back of the motoneuron occurs in even chronically diseased TG males, as all endplates are contacted by a motor nerve terminal (Kemp et al., 2011). Thus, deficits in axonal transport but not in neuronal loss or muscle atrophy correlate with motor dysfunction, suggesting that axonal transport dysfunction may contribute to early losses in motor function and ultimately to motoneuron death in SBMA.

This deficit in the retrograde labeling of motoneurons may be due to a number of factors, including dysfunction originating in the distal most aspect of the axon, the synaptic nerve terminal, where material is first taken up, sorted, and packaged into vesicles for either local recycling or retrograde transport to the motoneuronal cell bodies. Deficits could also originate in more proximal aspects of the axon. Transport of cargo in these two axonal compartments depends on different transport machinery, with local transport of cargo in the synaptic nerve terminal moved along actin by specialized myosin, whereas long-distance transport of cargo toward motoneuronal cell bodies is moved along microtubules by dynein and the motor-associated protein complex dynactin (Chevalier-Larsen and Holzbaur, 2006). Our first-level analysis of endosomal transport in living axons of diseased Tfm/TG mice indicated that endosomal flux, but not net velocity, was perturbed in Tfm/TG males, as found previously in chronically diseased myogenic males and acutely diseased myogenic females (Kemp et al., 2011). Thus, fewer labeled endosomes than normal move along the diseased axon per unit of time, offering an explanation for why fewer motoneurons in diseased Tfm/TG mice are retrogradely labeled after 12 h of CT transport. Such a deficit in flux suggests that the early endocytotic pathway in the nerve terminal is susceptible to non-cell-autonomous regulation, with disease signals originating in muscle perturbing some aspect of this presynaptic pathway, including possibly the uptake, local sorting, packaging/fusion, and/or movement of CT-containing cargo within the synaptic nerve terminal. Moreover, the nerve terminal exhibits the accumulation of neurofilament in SBMA patients, further linking the distal axon to defects in transport (Katsuno et al., 2006). Nevertheless, which of these processes is affected is not clear and would require direct monitoring of endocytosis in synaptic nerve endings to determine.

While net velocity was not affected by disease in Tfm/TG males, paradoxically, instantaneous velocity was, which is counter to previous findings reported for myogenic TG males (Kemp et al., 2011). A deficit in instantaneous velocity means that when endosomes are in motion, they move slower in diseased axons than in healthy axons, raising questions about whether AR in muscle can also cause defects in the retrograde motor dynein (Alper et al., 2013). A possible explanation for dynein dysfunction per se is suggested by a report by Ramzan et al. (2015). Using Cre-lox technology to drive expanded polyglutamine AR expression specifically in muscle fibers (MyoAR), they found reduced nitrotyrosine staining in motoneurons (Ramzan et al., 2015). These data suggest that a loss of oxidative homeostasis in motoneurons triggered by diseased muscle can, in turn, impair dynein. In fact, dynein heavy chain is reduced in the ventral spinal root of symptomatic AR97Q SBMA mice, but only in later stages of the disease (Katsuno et al., 2006).

The effect of disease on instantaneous velocity in Tfm/TG males is reminiscent of previous findings in a knock-in model of SBMA, also involving a deficit in instantaneous velocity (Kemp et al., 2011). While these previous data suggested that mutant AR expressed in motoneurons can cause defects in dynein/dynactin function in a cell-autonomous fashion, the current data suggest an additional hypothesis that endogenous (WT) AR in motoneurons of TG males may have protected against the toxic effects of muscle TG AR on instantaneous velocity. Knock-in males would not have this protection since every cell that expresses AR in this model expresses only the mutant *AR* allele. This suggestion is consistent with previous findings (Thomas et al., 2006) that endogenous WT AR can blunt the toxicity of a disease-causing TG AR in a different SBMA model. It is also possible that endogenous AR in muscle could have a protective role, but this scenario seems less parsimonious. Finally, while AR aggregates perturbed axonal transport in one SBMA cell model (Piccioni et al., 2002), this cannot explain the defects seen in the current TG model as AR aggregates do not occur in myogenic mice (Monks et al., 2007) or in axons of other SBMA models in which axonal transport defects have been found (Szebenyi et al., 2003; Katsuno et al., 2006).

We also found that retrogradely transporting endosomes in diseased axons had a higher overall incidence of trafficking perturbations, with more endosomes in the axons of Tfm/TG males showing reversals, stalls, and velocity changes than endosomes in the axons of healthy Tfm males (Fig. 3). Such “error” events reflect reduced processivity. An important regulator of processivity is the motor-associated protein dynactin (King and Schroer, 2000). In fact, dynactin is reduced in motoneurons of SBMA patients and in a mouse model of SBMA in which the disease allele is globally expressed and shows early defects in axonal transport (Katsuno et al., 2006), suggesting that it is likely to also be perturbed in the myogenic model. That transport of endosomes in axons of diseased Tfm/TG males is less efficient suggests that dynactin function is also perturbed in a non-cell-autonomous manner by diseased muscle. The run length of endosomes in axons of diseased Tfm/Tg males is also shorter than in axons of healthy Tfm axons, which is consistent with reduced processivity, though this difference is not significant, perhaps because of the increased error variance seen in healthy Tfm controls. Nonetheless, together these data suggest that signals from diseased muscle can instigate defects in the core transport machinery involving the retrograde motor and its associated proteins, although the effect of diseased muscle on retrograde transport seems to lie predominantly in the motor synapse where cargo is first taken up and packaged. While the current study represents a small step toward recognizing the potential role of non-cell-autonomous regulators of axonal transport, this possibility is sufficiently important to merit further investigation.

The fact that instantaneous velocity, but not net velocity, is perturbed in Tfm/TG diseased males suggests that compensation occurred. One possible explanation is that while reversals are more common in diseased axons, perhaps the distance traversed in the wrong direction is less in diseased axons than in healthy axons. Regardless of the explanation for the apparent discrepancy in instantaneous versus net velocity, the current findings raise the possibility that dynein and dynactin, which are essential in the retrograde transport of cargo, are each susceptible to non-cell-autonomous regulation by the muscle. While there is precedence that both dynein and dynactin are impaired in models of SBMA (Szebenyi et al., 2003; Katsuno et al., 2006), the current data are the first to suggest that such defects in motor proteins could be caused via a non-cell-autonomous mechanism.

How might the action of a toxic AR in muscle perturb axonal transport in the motoneurons? One attractive scenario is the loss of muscle-derived trophic factors. Skeletal muscle releases many kinds of trophic factors that can act on receptors in motor nerve terminals to promote healthy cell function. Diseased muscle from various SBMA mouse models exhibits deficits in multiple trophic factors, including brain-derived neurotrophic factor (BDNF), glial cell line-derived factor, insulin-like growth factor 1, neurotrophin (NT)-4, and vascular endothelial growth factor (VEGF) (Sopher et al., 2004; Yu et al., 2006; Monks et al., 2007; Mo et al., 2010; Halievski et al., 2015). Moreover, replenishing the supply of such neurotrophic factors can reverse disease-related deficits in axonal transport in models of motoneuron/neuromuscular disease. For example, delivery of ciliary neurotrophic factor, BDNF, or NT-3 to muscle improves retrograde transport in the progressive motor neuronopathy mouse model (Sagot et al., 1998). Additionally, treating diseased muscles of myogenic TG SBMA females with VEGF reverses a disease-related deficit in retrograde labeling of motoneurons and endosomal flux (Kemp et al., 2011). These data are compelling precedence for a cause–effect relationship between muscle-supplied neurotrophic factors and retrograde transport in motoneurons.

**Figure 4.**
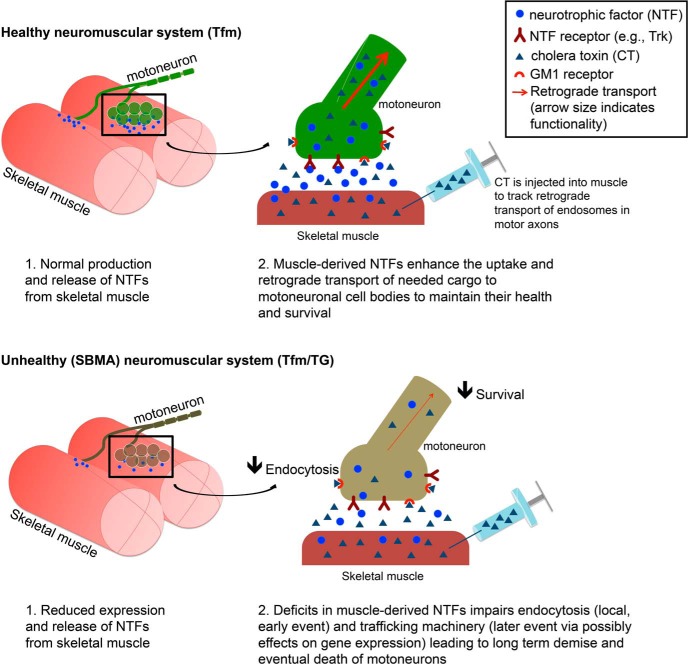
Proposed model of the non-cell-autonomous influence of muscle on motoneuronal axonal transport. In the healthy neuromuscular system, neurotrophic factors produced and released from skeletal muscle support motoneuronal health and survival. SBMA skeletal muscle is deficient in neurotrophic factors, which may lead to reduced endocytosis in motoneurons, which may manifest as reduced retrograde transport of cholera toxin and reduced endosomal flux, as observed in the present study. Therapies aimed at increasing muscle-derived neurotrophic factors may remedy defective axonal transport and ameliorate disease symptoms.

Loss of such trophic support from target musculature could lead to defective transport in at least two ways. First, lack of trophic factor signaling might mean a loss of survival signaling, leaving the cell deprived of needed resources for proper maintenance of axonal transport (Chowdary et al., 2012). Alternatively, local neurotrophic signaling may be needed for endocytosis per se, since VEGF can reverse a deficit in flux (Kemp et al., 2011). Internalization of the neurotrophin-bound Trk receptor requires PI3K signaling to recruit Rab5 (Christoforidis et al., 1999; for review, see Harrington and Ginty, 2013). Since internalization and sorting of the CT receptor GM1 also depends on Rab5 (Pelkmans et al., 2004), neurotrophic factors could enhance endocytosis through this pathway. Once internalized, Trk signaling also leads to the phosphorylation of dynein intermediate chains through the MAPK/ERK pathway to enhance the binding of dynein to Trk-activated signaling endosomes (Mitchell et al., 2012). Defects in this pathway could explain the deficit in flux as well as the reduced efficiency of transport in diseased axons. In short, muscle-derived neurotrophic factors are a strong candidate for how diseased muscle might disrupt the uptake and/or retrograde transport of cargo in motoneuronal axons of SBMA mice (Fig. 4, proposed model). While data are scant, the relevance of these data to SBMA in humans is likely (Katsuno et al., 2006).

While considerable attention has recently been redirected to muscle, recent work also supports the idea that mutant AR acting in motoneurons contributes to SBMA pathogenesis (Ramzan et al., 2015; Sahashi et al., 2015). Given that both motoneurons and muscle normally express AR, and that motoneurons and muscles are interdependent, it would not be surprising if the symptoms of SBMA reflect a complex interplay of mutant AR acting in both motoneurons and skeletal muscles, if not in other cell types too. The important task before us is to better understand where and how mutant AR acts to cause disease and to sort out the primary effects of AR from the secondary effects of disease. AR acting in muscle to impair axonal transport may be one such early pathogenic event leading to widespread neuromuscular dysfunction.

## Synthesis

The decision was a result of the Reviewing Editor Douglas Bayliss and the peer reviewers coming together and discussing their recommendations until a consensus was reached. A fact-based synthesis statement explaining their decision and outlining what is needed to prepare a revision is listed below. The following reviewer(s) agreed to reveal their identity: Maria Pennuto

Two reviewers have provided comments on this manuscript and both agree that this work will be important to understanding SBMA, and associated non-cell-autonomous defects of axonal transport. However, some major issues with the manuscript remain to be addressed.

In short, it is expected that you should provide data from all experimental groups (i.e., including WT and TG) that addresses either or both of:

1. Analysis of retrograde transport at shorter time points, like 1-2 days after treatment with testosterone;

2. Analysis of phenotype for recovery after stopping testosterone treatment.

For the issue of controls, there are concerns regarding comparisons between T-treated TG female and Tfm/TG males that are discussed in the manuscript. In your rebuttal, you mention other confounds in making the recommended comparisons between TG and TG/Tfm mice as controls. In light of this, a frank discussion should be included in the Discussion that explicitly acknowledges the shortcomings of these different control data and associated arguments.

Reviews

Advances the Field

The major advancement in the field is the observation that non-cell-autonomous defects of axonal transport are detected in SBMA

The study gains insight into the pathogenesis of Kennedy's disease.

Comments to the Authors

The authors have not addressed specific concerns of this reviewer, including the analysis of retrograde transport at shorter time points, like 1-2 days after treatment with testosterone. Even if they claim that female tg mice are comparable to male tfm/tg mice, and in these females they have shown defects in axonal transport upon short time points of treatment, it remains to be established whether this effect is cell autonomous. Otherwise, the use of this experimental paradigm is meaningless. Lack of all the appropriate controls limits the interpretation of the results. If WT mice were analyzed, and 4 experimental groups were used, it would be nice to see the data for the all 4 genotypes. Even analysis of phenotype for recovery after stopping testosterone treatment would have been interesting, even if something like that has been done in female TG mice. Females express endogenous AR and have circulating testosterone, even if at very low doses, which is the reason why a deeper analysis of the phenotype of the tfm/tg mice would have been worth to do to investigate the non-cell-autonomous component inherent to axonal transport.
